# Editorial: Artificial intelligence based diagnostics for neurological disorders

**DOI:** 10.3389/fnhum.2023.1287959

**Published:** 2023-10-05

**Authors:** Enas Abdulhay, Oliver Faust, Gustavo Ramirez

**Affiliations:** ^1^Department of Biomedical Engineering, Jordan University of Science and Technology, Irbid, Jordan; ^2^Faculty of Science and Engineering, School of Computing and Information Science, Anglia Ruskin University, Cambridge, United Kingdom; ^3^Department of Telematics, University of Cauca, Cauca, Colombia

**Keywords:** artificial intelligence, diagnostics, neurological disorders, features, deep learning

The future of the world is going to be dominated by the artificial intelligence (AI) viewpoint in all areas of medical research. Neurological disorders are complex types for diagnosis, particularly at the early stage. Early detection can support the patient in innumerable ways to get cured. With these in mind, the topic AI-based diagnostics for neurological disorders was proposed as a special section. A total of 11 articles were received in the special section. After rigorous review, 4 papers were accepted. A short synopsis of these 4 accepted papers is presented as follows:

Zhao et al. presented a mechanical learning model for the prediction of sepsis. They used multivariable logistic regression analysis by employing more than 2,000 pieces of data from sepsis patients. Nine clinically significant features were extracted and analyzed. They obtained a value of 0.743 for the area under the curve (AUC) related to the AI-based detection of sepsis. This could provide a significant application for a clinical set-up in the future.

Kim et al. aided a machine learning model to predict brain amyloid pathology in pre-dementia Alzheimer's disease. They employed the EEG and extracted and analyzed 152 features through a genetic algorithm to identify optimal combinations of features. Using appropriate standard classifiers, they achieved a highest accuracy of 88.6%.

Sun et al. demonstrated a novel deep learning approach for Alzheimer's disease (AD) diagnosis based on eye-tracking data. Eye-tracking-based diagnosis is a recent trend that presents an easy method for diagnosis in the future. They used a nested autoencoder network to extract the eye movement features and a weighted adaptive network layer for feature fusion to feed them to a binary classifier with four-fold validation. They achieved a highest classification accuracy of 85% in AD detection, which is higher than the methods available in the present literature.

Finally, Hsieh et al. administered a seed correlation analysis for ADHD diagnosis. A two-step hierarchical analysis method was used to extract the functional connectivity features. Furthermore, they were evaluated by linear classifiers. The features proposed resulted in a success rate of 83.24% in detecting ADHD.

## Author contributions

EA: Writing—original draft, Writing—review and editing. OF: Writing—review and editing. GR: Writing—review and editing.

